# Understanding competency of nursing students in the course of case-based learning in Cambodia: a convergent mixed method study

**DOI:** 10.1186/s12912-023-01420-8

**Published:** 2023-08-11

**Authors:** Kyoko Koto-Shimada, Rogie Royce Carandang, Akira Shibanuma, Junko Kiriya, Ken Ing Cherng Ong, Sokneang Touch, Virya Koy, Masamine Jimba

**Affiliations:** 1 Department of Community and Global Health, School of International Health, Graduate School of Medicine, The University of Tokyo, 7-3-1 Hongo, Bunkyoku, Tokyo, 113-0033 Japan; 2 Department of Public Health Sciences, University of Connecticut School of Medicine, 263 Farmington Avenue, Farmington, Storrs, Connecticut, 06030-6325 USA; 3 Department of Human Resource Development, Ministry of Health Cambodia, No:80, Samdach Penh Nouth Blvd (289), Sankat Beoungkak 2, Tuol Kork District, Phnom Penh, Cambodia; 4 Department of Hospital Service, Ministry of Health Cambodia, No:80, Samdach Penh Nouth Blvd (289), Sankat Beoungkak 2, Tuol Kork District, Phnom Penh, Cambodia

**Keywords:** Associate, Academic training, Case-based learning, Cambodia, Competency, Convergent mixed method study, satisfaction

## Abstract

**Background:**

In the last decade, nursing education has begun to reform to competency-based education worldwide, including in low-and middle-income countries. Case-Based Learning (CBL), an approach to delivering competency-based education, contributes to acquiring critical thinking competency, problem-solving, higher knowledge, professional value and attitude. However, it needs to be taught in a culturally appropriate manner. In Cambodia, CBL was initiated in a classroom and clinical practicum by faculty and preceptors who graduated from the upgrading course. This study examined the factors associated with the competency level of nursing students, explored the practice and perceptions of teaching–learning activities among students, faculty members and preceptors and assessed the coherence of qualitative and quantitative findings.

**Methods:**

This was a convergent, mixed methods study. Data were collected from eight educational institutions for quantitative and qualitative studies and seven hospitals for qualitative studies. From June to September 2019, a cross-sectional survey of nursing students in the third year of the three-year programme (n = 719), eight focus group discussions (FGDs; n = 55) with 6–8 members and 15 FGDs with faculty (n = 38) and clinical preceptors (n = 37) with 4–7 members were conducted to elicit the teaching–learning experience and perceptions. Multiple linear regression was performed to investigate the factors associated with student competency. Moreover, the study conducted thematic content analysis on the qualitative data. The integrated analysis was presented as side-by-side joint displays.

**Results:**

First, the quantitative and qualitative findings confirmed each other ’s CBL learning experiences. Students had higher levels of nursing competencies if they had CBL experiences, both in the classroom and clinical practicum, both in a group manner. Next, the quantitative and qualitative findings complemented students’ academic satisfaction with *the teaching by faculty members and preceptors*. Finally, the quantitative and qualitative findings were expanded to explain students’ academic satisfaction with the *programme*.

**Conclusions:**

The finding of CBL experiences in a group and students’ satisfaction with faculty members’ and preceptors’ teaching improved nursing students’ competency development. Meanwhile, students’ satisfaction with the design and delivery of the educational programme provides implications for policy level to narrow the theory and practice gaps in low- and middle-income countries.

**Supplementary Information:**

The online version contains supplementary material available at 10.1186/s12912-023-01420-8.

## Background

Delivering competency-based education is a critical challenge of pre-service education for nurses [1]. Specifically, competency-based education is an outcome-based approach that addresses learning in the cognitive, psychomotor and affective domains [1]. In the last decade, nursing education has begun to reform toward competency-based education worldwide, including low- and middle-income countries [2]. Scholars identify its effects in improving the knowledge acquisition, skill performance and behavioural changes of nursing students [3, 4]. However, evidence is limited about measuring the outcome of competency-based education and identifying new approaches for developing the abilities of faculty members and preceptors in resource-limited settings [5].

Case-Based Learning (CBL) is an approach for delivering competency-based education, which contributed to the acquisition of critical thinking competency [6], problem-solving [7], high levels of knowledge [8], self-confidence and better preparation for practice settings [9]. The components of CBL are based on real-world situations, group learning, student-directed solutions to problems and the role of teachers as facilitators in a classroom teaching setting [10]. In Cambodia, CBL was newly introduced by the faculty members and preceptors who experienced CBL learning during an upgrading course in Thailand [11, 12]. However, CBL must be taught as culturally appropriate methods [13]. For instance, in certain cultures, students tend to feel stressed when asking questions to teachers due to the traditional seniority system in Asia. This notion is becoming an obstacle to the acquisition of critical thinking skills [14]. However, few studies demonstrate how CBL has benefited nursing competencies in Southeast Asian countries.

Moreover, the academic satisfaction of students is an attribute of competency development. Nurse educators play an essential role in providing a supportive learning environment and mentorship [15]. Nursing students with high levels of satisfaction with quality of education produce better learning outcomes when an educational institution and a hospital nurture a positive working relationship [16]. However, the concept of *saving face* promotes the high evaluations of students on teaching and learning activities in the Southeast Asian culture [17]. Therefore, quantitative and qualitative assessments could provide an accurate view of the satisfaction of students.

Cambodia is a post-conflict country that has undergone a significant transition during the 2010s. Within this period, political stability and economic growth have improved the health of the Cambodian population. The Cambodian Ministry of Health (CMOH) has strengthened its health system to overcome these challenges. Moreover, it has planned to revise the current nursing curriculum to a competency-based one to prepare nurses who fit the regional standard under the Association of Southeast Asian Nations (ASEAN) Mutual Recognition Arrangement [18]. Thus, improving the capacity of nurses through educational programmes has been one of the priorities of the CMOH. Moreover, the academic degrees of nursing leaders have been upgraded to ensure competency in nursing education and service [19]. Furthermore, the number of faculty members who graduated from a four-year programme has increased from 10 to 2010 [20] to 68 in 2019 in public educational institutions (School Statistics, 2019). Therefore, upgrading the academic background of faculty members has exerted a favourable effect on the improvement of teaching and learning activities [11] such as the introduction of CBL [12]. However, the outcome of these improved teaching and learning activities has yet to be assessed sufficiently. This study examined the factors associated with the competency level of nursing students, explored the practice and perceptions of teaching–learning activities among students, faculty members, and preceptors and assessed the coherence of qualitative and quantitative findings [21].

## Methods

### Design

**Fig. 1:**
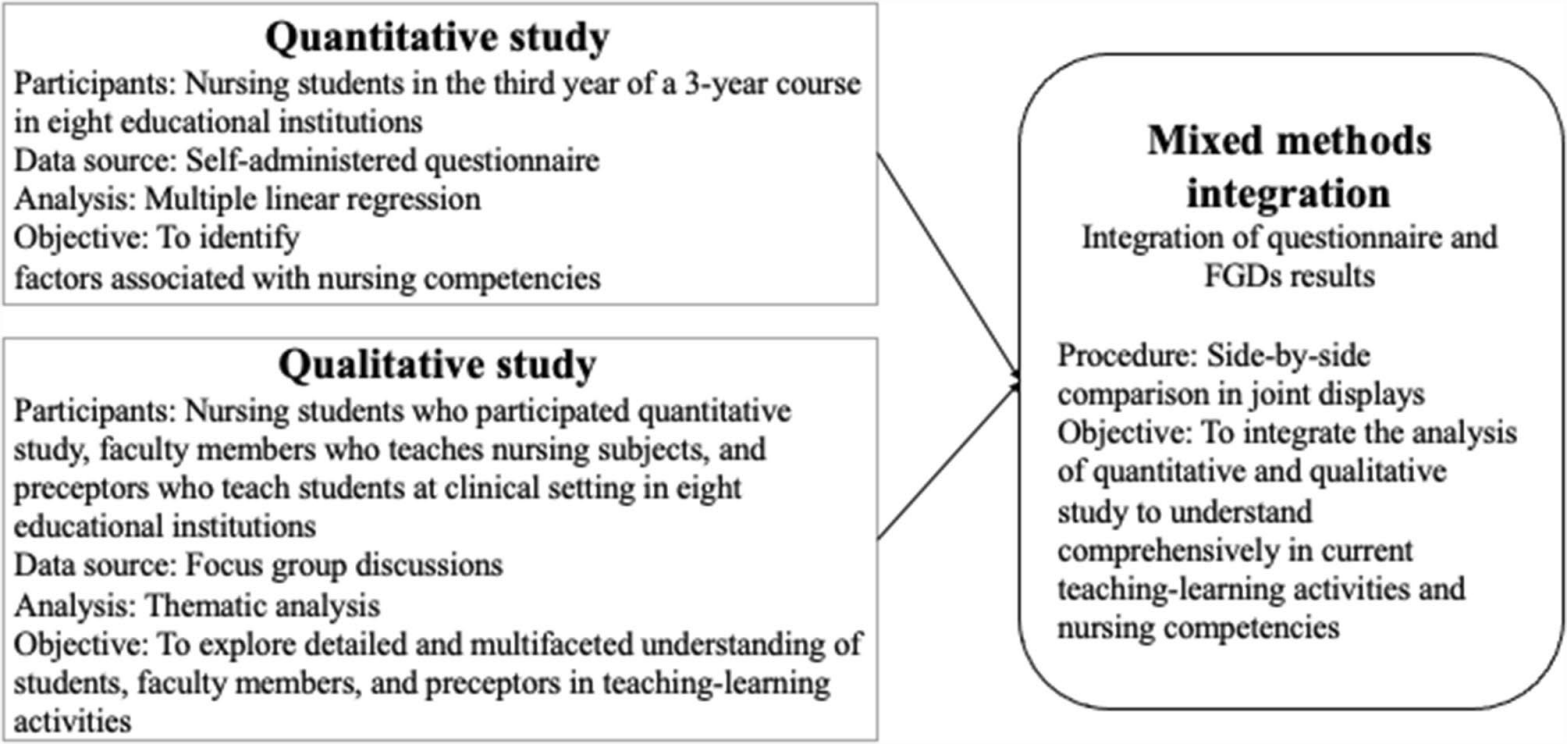
Outline of research framework

The study intended to develop a broad understanding of the nursing competency of students in Cambodia by comparing the quantitative and qualitative results [22]. Figure 1 outlines the research framework. The study employed a convergent mixed methods approach, including the simultaneous collection of quantitative and qualitative data [23], which enabled rapid data collection nationwide [22]. The quantitative study was applied to examine the factors associated with the level of nursing competency. Meanwhile, the qualitative study explored the detailed and multifaceted understanding of students, faculty members and preceptors about teaching and learning activities. For example, the cultural norm of *saving face* in the Southeast Asian culture could result in the high rates of quantitative evaluation by students on teaching–learning activities [17]. Supplementary material Table 1 depicts that the contents of the questionnaire and the topic guide for the FGDs were matched to examine the manner in which the datasets diverge or complement one another [21]. Accordingly, a side-by-side comparison displayed the final integration [24].

### Quantitative study

#### Participants

In Cambodia, a total of 19 educational institutions, including six public and thirteen private ones, operate a three-year nursing programme. The inclusion criteria of the study were students in the third year of the three-year nursing programme and the final year of an upgrading course for primary nurses. Data were collected from students in eight educational institutions, including all six public institutions (supplementary material Fig. 1). The initial research plan included three private educational institutions with more than 20 students in the capital city. However, only two private institutions agreed to participate in the study.

In order to detect an effect size of Cohen’s d = 0.3 with 80% power (alpha = 0.05, two-tailed), G*Power suggested we would need 176 participants per group (N = 704) in an independent samples t-test. The smallest effect size of interest was set to d = 0.3 [25]. Given the dropout rate, we recruited all 840 students who met the inclusion criteria at the participating educational institutions. The authority of each target institution appointed a faculty coordinator to support the study. They informed the students of their participation prior to data collection.

#### Data collection and materials

**Fig. 2:**
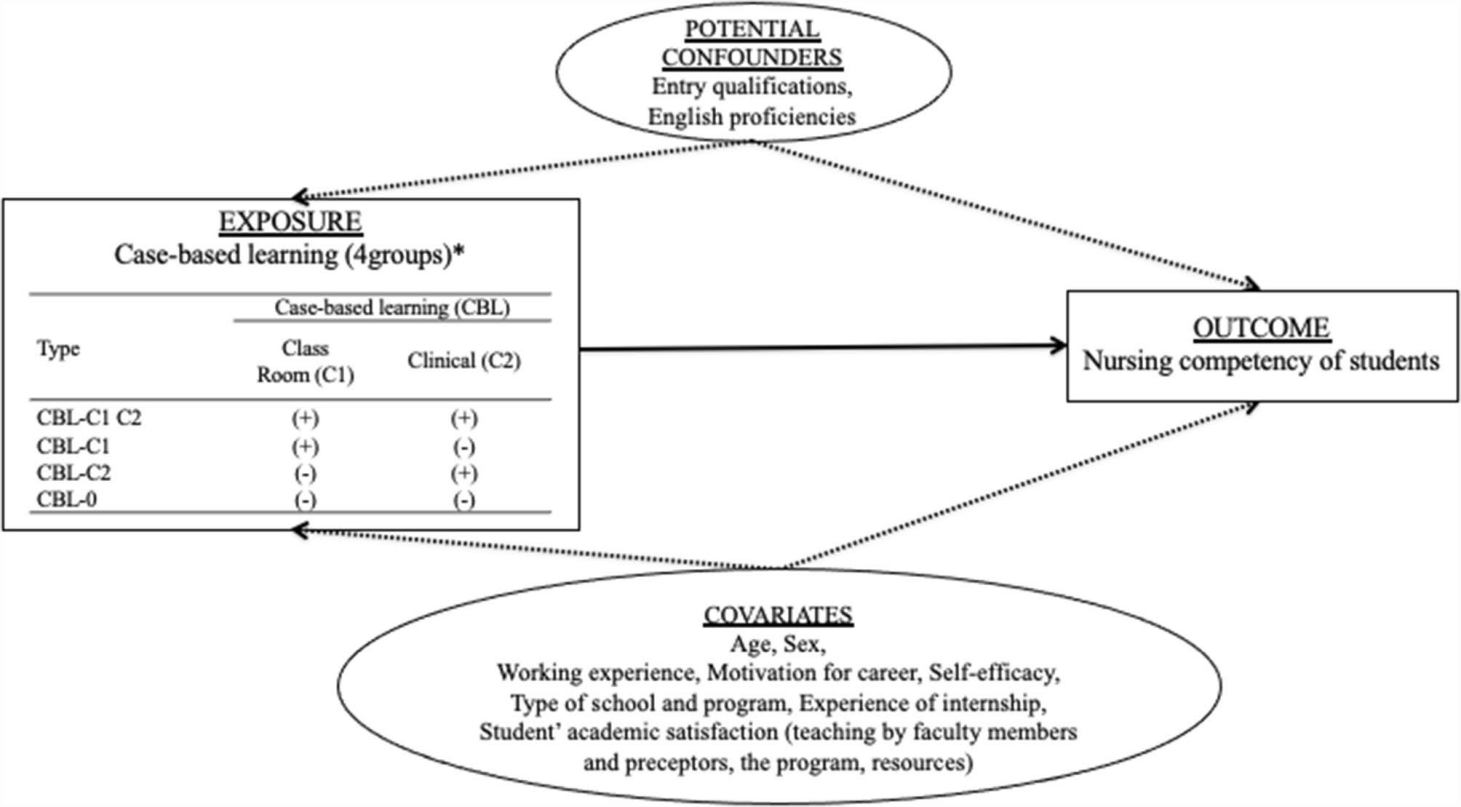
Conceptual framework for the quantitative study. *CBL-C1C2 group: students who experienced case-based learning in the classroom (C1) and clinical practicum (C2). CBL-C1 group: students who experienced case-based learning only in the classroom (C1). CBL-C2 group: students who experienced case-based learning only in clinical practicum (C2). Lastly, CBL-0 group students without experience of case-based learning in any of the two settings

A cross-sectional survey was conducted among the nursing students between June and August 2019, using a self-administered questionnaire (Supplementary material Table 2). The questionnaire was developed using a conceptual framework for the quantitative study as shown in Fig. 2. We hypothesised that the existing CBL experiences in the classroom and clinical practicum would be associated with the nursing competencies of students.

For the outcome variable, the nursing competency of students was measured using the 39 items from the Competency Inventory for Nursing Students [26]. This inventory was developed in Taiwan and consists of six domains, namely, (1) ethics and accountability, (2) general clinical skills (e.g., application of the nursing process), (3) lifelong learning, (4) clinical biomedical science, (5) caring and (6) critical thinking and reasoning. These domains are similar to those in the core competencies framework for nurses in Cambodia [27]. The items of the original scale are rated using a seven-point Likert scale that measures the achievement of competency. However, asking the frequency for each competency item was recommended because it was the most accurate but least threatening method of assessing competency development [28]. Thus, the rating was modified from achievement to frequency. The response options for all the items were 1 = *not at all*, 2 = *seldom*, 3 = *occasionally*, 4 = *sometimes*, 5 = *frequently*, 6 = *nearly always*, and 7 = *always*. Possible scores range from 39 to 273 with high scores indicating high frequencies of competency. Two items from the original version are revised, and items on the code of ethics and nursing regulations are merged into one statement. Consequently, the item that gave equal shared medical resources, which was outside the scope of nursing service in Cambodia, is omitted. Cronbach’s α for this study was 0.93.

For the exposure variable, the study assessed the existing experience of students with CBL as shown in Fig. 2. The students were categorised into four groups, namely, (1) CBL-C1C2 group: students who experienced CBL in the classroom (C1) and in the clinical practicum (C2); CBL-C1 group: students who experienced CBL only in the classroom (C1); CBL-C2 group: students who experienced CBL only in the clinical practicum (C2); and (4) CBL-0 group: students without experience of CBL in any of the two settings.

The sociodemographic variables included age, sex, type of educational programme, plan after graduation, working experience, and internship experience. This study included high school grade and English proficiency as potential confounders. For high school grades, the study employed five levels according to the high school system of the Cambodian government, which were grouped into three, namely, more than good, satisfactory, and limited achievement. English proficiency facilitates access to various sources, such as documents or the Internet, because of the limited teaching–learning materials in the Khmer language in Cambodia [12]. Self-assessed English proficiency was grouped into three, namely, more than moderate, low, and very low.

In addition, this study measured the level of satisfaction of the students as a covariate using the 44-item Undergraduate Nursing Student Academic Satisfaction Scale (UNSASS) [29]. The scale consists of four subscales that evaluate satisfaction with (1) the teaching by faculty members, (2) teaching by preceptors, (3) the programme and (4) resources. Nursing students indicated the extent to which they agreed with each statement using a five-point Likert-type scale ranging from 1 (*strongly disagree*) to 5 (*strongly agree*). The total UNSASS scores ranged from 44 to 220 with high scores indicating high levels of satisfaction. Cronbach’s α for this study was 0.94.

We obtained approval from the original authors to use and translate the Competency Inventory for Nursing Students and Undergraduate Nursing Student Academic Satisfaction Scale for the study. First, a questionnaire was developed in English and translated into Khmer. For the translation, translation and cultural adaptation were considered [30]. Supplementary material Fig. 2 explains the flow of the translation and pilot testing. Two research assistants independently prepared the drafts in Khmer (forward translation), compared their translations and combined them into one version (reconciliation). Another professional translator, who was blinded to the questionnaire, translated it back into English (back-translation). Two research assistants translated it again until the back-translated version matched the meaning of the original version (reconciliation). Two research assistants and the first author (KKS) conducted two pilot tests to assess the understanding of the nursing students of the questions. The questionnaire was then corrected accordingly prior to conducting the actual survey.

#### Data analysis

The study used descriptive statistics to summarise the sociodemographic characteristics of the participants. Multiple linear regression was performed to investigate the factors associated with the nursing competency of students. Analysis included exposure, covariates, and potential confounders. After the regression analyses, any variable with a variance inflation factor of more than 10.0 was excluded. Subsequently, hierarchical regression analysis was performed to ascertain the predictors of the subscales of academic satisfaction. Model 1 adjusted for the teaching by faculty members, model 2 for the teaching by faculty members and teaching by preceptors, model 3 for the teaching by preceptors and the programme, model 4 for the teaching by faculty members, teaching by preceptors and the programme and model 5 adjusted for all subscales, including resources. Statistical analyses were performed using Stata/IC 13.1 (College Station, Texas, USA). Statistical significance was set at 0.05.

### Qualitative study

#### Participants

The participants of the study were students, faculty members, and preceptors. The inclusion criteria for students were the same as those for the quantitative study. In addition, they were selected based on their responses in the quantitative study for further qualitative explanations on academic satisfaction or CBL experiences: (1) students who chose a career apart from being a nurse after graduation or (2) students with CBL experience in the current educational institution. Six to eight students were invited to participate as an optimal number for the FGD. The faculty coordinator informed the students of their participation.

The inclusion criteria for faculty members and preceptors were: (1) faculty members who taught one of the seven nursing subjects for more than one year at one of the eight educational institutions and (2) preceptors who worked with students during the practicum in one of five clinical areas. After selecting the seven faculty members and five preceptors who met the abovementioned criteria, the faculty or hospital coordinator invited the relevant participants to come to a specified room in their educational institutions or hospitals.

#### Data collection and materials

A topic guide was developed for FGDs based on the objectives and theoretical framework of the study. Sample questions included the following (Supplementary material Table 3): (1) How did you learn/teach the nursing process in the classroom and clinical setting? (2) What kind of support did you receive or provide from/to faculty members and preceptors or students? (3) What is your opinion about the role of faculty members and preceptors in supporting students? (4) What challenges do you face in learning? Two research assistants who are fluent in English and Khmer, the local languages, and the first author (KKS) conducted the FGDs. Each FGD lasted for approximately 90–100 min. Interviews were conducted until data saturation was reached, and no new codes emerged. Data were collected from June to September, 2019. The first author (KKS) maintained contact with a few faculty members and preceptors from previous studies [11, 12].

Face validity testing was conducted with author (VK), who holds a PhD in nursing and is fluent in English and Khmer. In addition, pilot testing was performed with fourth-year students in a four-year nursing programme, and the topic guide was modified accordingly. The two research assistants and the first author familiarised themselves with the data by meeting to discuss and compare the field notes after each FGD.

#### Data analysis

All interviews were audio-recorded and transcribed verbatim in Khmer. After the two research assistants translated the transcripts into English, the first author checked the transcripts to identify any discrepancies from the field notes. The transcripts were subsequently analysed using the thematic analysis approach, which is a useful method for examining the perspectives of various research participants [31]. Two authors (KKS and RRC) read the transcripts, generated initial codes, searched for categories, produced a list of themes and interpreted the results. They validated the coding and themes through continuous dialogue among the researchers. Furthermore, feedback was provided to interviewees by summarising their findings prior to the finalisation of the main themes and categories. They confirmed the results and provided valuable feedback, which helped to further refine the present findings.

#### Mixed methods integration

The convergent mixed method study integrated the quantitative and qualitative findings by matching the data on the experience of students with CBL, academic satisfaction with the support of faculty members and preceptors and academic satisfaction with the programme, as shown in supplementary material Table 1. The integrated analysis was presented as a side-by-side joint display. The fit was assessed between the quantitative and qualitative findings as confirmation, complementarity, expansion, or discordance [24].

## Results

### Quantitative study

A total of 722 students (86.0%) participated, and each survey lasted for 30–45 min. The study then analysed data from 719 students, excluding three students, because they did not complete the questionnaire.

Table 1 presents the sociodemographic characteristics of the students. Out of 719 students, 53% were women. The mean age was 23.1 years (standard deviation [SD]: 3.4). A total of 56.5% reported their self-assessed English proficiency to be higher than moderate. Moreover, 47.7% reported high school grades as “limited achievement,” 95% planned to work as a nurse, and 73.4% reported continuing their study in health or other sectors.

**Table 1:** Characteristics of the participants

Characteristic	Total (N = 719)	
	n	%
**Sex, men**	337	46.9
**Age, mean (SD)**	23.1	(3.4)
**English proficiency**		
>Moderate	406	56.5
Low	262	36.4
Very low	51	7.1
**High school grade**		
Good and higher	167	23.2
Satisfactory	209	29.1
Limited achievement	343	47.7
**Previous working experience**	177	24.6
**Length of working experience in months, mean (SD)**	42.5	(35.2)
**Type of school (public)**	568	79.0
**Type of course, upgrading course from primary nurses**	90	12.5
**Internship (finished)**	230	32.0
**Plan after graduation**		
Work as nurse	683	95.0
Continue to study	528	73.4
Neither study nor work in nursing	36	5.0

Table 2 presents the factors associated with the nursing competencies of the students. After conducting the regression analyses, variance inflation factor values was less than 4.0, which was acceptable. The proportion of students according to CBL experience resulted in the highest percentage for the CBL-0 group (47.6%) followed by the CBL-C1C2 group (42.7%). Hierarchical regression analysis with all subscales of the UNSASS (Model 5) explained 31.3% of variance in nursing competencies. The CBL-C1C2 group was positively associated with a high frequency of competencies (B = 5.19; 95% CI = 1.25, 9.13) compared with that those of the CBL-0 group. High school grades that were *satisfactory* (B = 8.62; 95% CI = 4.61, 12.63) and *higher than good* (B = 6.42; 95% CI = 2.01, 10.84) were positively associated with a high frequency of competencies with that of *limited achievement*. The level of English proficiency at *higher than moderate* (B = 8.90; 95% CI = 2.28, 15.51) was positively associated with a high frequency of competency compared with that of *very low*. The subjective satisfaction of the students with *the teaching by faculty members* (B = 0.56; 95% CI = 0.21, 0.90) and *programme* (B = 1.21; 95% CI = 0.65, 1.77) were also positively associated with a high frequency of competencies.

**Table 2:** Factors associated with nursing competencies (N = 719)

Variables	B^a^	95% CI for B	β^b^	*p*-Value
**Case-based learning** ^*^ **(vs. CBL-0)**				
CBL-C2	2.82	(− 4.41, 10.04)	0.03	0.445
CBL-C1	−2.63	(− 12.78, 7.51)	−0.02	0.610
CBL-C1C2	5.19	(1.25, 9.13)	0.10	0.010
**Age**	−0.10	(− 0.92, 0.72)	−0.01	0.813
**Sex (**vs.**man)**				
Woman	–1.58	(− 5.14, 1.98)	−0.03	0.385
**High school grade (vs. limited achievement)**				
Satisfactory	8.62	(4.61, 12.63)	0.16	< 0.001
>Good	6.42	(2.01, 10.84)	0.11	0.004
**English proficiency (vs. very low**)				
Low	4.23	(− 2.40, 10.86)	0.05	0.211
>Moderate	8.90	(2.28, 15.51)	0.10	0.008
**Previous working experience (vs. none)**				
<12 months	7.01	(1.22, 12.80)	0.09	0.018
13–48 months	10.03	(0.19, 19.88)	0.07	0.046
>49 months	4.11	(− 8.70, 16.92)	0.02	0.529
**Type of program (vs. upgrading course)**				
Three-year program	3.05	(− 8.07, 14.17)	0.02	0.590
**Type of school (vs. public school)**				
Private school	0.17	(− 5.78, 6.11)	0.00	0.956
**Internship (vs. not done yet)**				
Finished	3.50	(− 0.59, 7.59)	0.06	0.093
**Self-efficacy**	1.56	(1.10, 2.02)	0.24	< 0.001
**Satisfaction**				
Teaching by faculty members	0.56	(0.21, 0.90)	0.15	0.002
Teaching by preceptors	0.28	(− 0.10, 0.66)	0.07	0.149
Program	1.21	(0.65, 1.77)	0.20	< 0.001
Environment	−0.11	(− 1.57, 1.36)	−0.01	0.886

R2 (%) 31.3.

^a^Unstandardized coefficients.

^b^Standardized coefficients.

*CBL-C1C2 group: students who experienced case-based learning (CBL) in the classroom (C1) and clinical practicum (C2). CBL-C1 group: students who experienced CBL only in the classroom (C1). CBL-C2 group: students who experienced CBL only in clinical practicum (C2). Lastly, CBL-0 group: students without experience of CBL in any of the two settings.

### Mixed methods findings

For qualitative study, supplementary material Table 4 provides the characteristics of the students, faculty members and preceptors who participated in the FGDs.

Table 3 presents the key findings between quantitative and qualitative studies.

**Table 3:** Side-by-side joint display of the key findings between quantitative and qualitative studies

Key finding	Quantitative findings (Multiple regression analysis)	Qualitative findings (Themes)	Meta inference
CBL experience and nursing competency development	The CBL-C1C2 group was positively associated with a high frequency of competencies (B = 5.19; 95% CI = 1.25, 9.13) compared with the CBL-0 group.	Learning and teaching experiences of CBL in a classroom and a clinical practicum	**Confirmation** Teaching through CBL in group work was introduced in the classroom and the clinical practicum on a pilot basis, which explains the improvements observed in the nursing competency scores.
Satisfaction with the teaching by faculty members and preceptors and nursing competency development	The subjective satisfaction of the students with the *teaching by faculty members* (B = 0.56; 95% CI = 0.2, 0.9) was positively associated with a high frequency of competencies.	Students’ experience and needs during clinical practicum through the support of faculty members and preceptors - Limited support from faculty members - Need for active support from faculty members and preceptors	**Complementarity** A few students were dissatisfied with the lack of support from the faculty members during the clinical practicum. Meanwhile, those who were satisfied exhibited improved nursing competency scores.
The satisfaction of the students with the program and nursing competency development	The subjective satisfaction of the students with the program (B = 1.21; 95% CI = 0.7, 1.8) was positively associated with a high frequency of competencies.	Inconsistencies between theory and practice - Clinical nursing techniques - Implementation of the nursing process	**Expansion** Inconsistencies in the program (e.g., clinical nursing technique and process) explain the satisfaction with the program through the nursing competency scores.

### CBL experience and nursing competency development

The quantitative data indicated that the CBL-C1C2 group was positively associated with a high frequency of competencies compared with the CBL-0 group, who never experienced CBL in any of the two settings. Alternatively, the qualitative data described as a confirmatory quote as learning and teaching experiences of CBL in the classroom and in the clinical practicum from students, faculty members and preceptors. Students responded classroom teaching as being lecture-based or as a set of lectures and technical practices. Furthermore, they added the development of a nursing care plan in group work as an example of CBL in the classroom.

‘*Teacher gave the case scenario for students to learn in class by using the nursing care plan form. After that, we presented in class, receive peer evaluation, and teacher’s feedback’.* (Student, R3-S3)

The faculty members and preceptors presented CBL as a set of lectures, demonstrations and technical practices. Group presentation, peer evaluation and feedback were included in the CBL assessment.

‘*Faculty members added a clinical case assignment for students to apply at the educational institution and hospital’.* (Faculty, R4-S3)

*‘We assigned the case study to students using the clinical case assignment form developed by the school’.* (Preceptor, R3-H2)

### Satisfaction of students with teaching by faculty members and preceptors and nursing competency development

The quantitative data indicated that student satisfaction with classroom teaching was positively correlated with high levels of competency. The qualitative data complemented as the experience of limited support from faculty members and need for active support from faculty members and preceptors during the clinical practicum.

Students emphasised the limited follow-up in the clinical practicum provided by faculty members, such as accompanying them on their first day, spot-checking (i.e. attendance and discipline management) and focusing on evaluation. Faculty members also admitted minimal involvement in the clinical practicum, which included arranging the clinical practicum site, visiting on the first day to introduce the students and spot-checking attendance and discipline.

*‘Teachers only came to the hospitals with us on the first day to do the orientation. They (teachers) didn’t monitor our clinical skills at the bedside’. (Student, R3-S6)*

*‘My title was a clinical instructor, but I just spent 30 percent of my task at clinical practicum’. (Faculty, R3-S2)*

The students expressed a greater need for psychological support. When they experienced difficulties in addressing patients and families in critical cases, they tried to cope by asking for support from faculty members and preceptors or by discussing among themselves. They also expected faculty members and preceptors to work as a team. Meanwhile, preceptors responded that they had an overall task during their clinical practicum. They provided orientation, assigned cases to students, demonstrated and evaluated clinical nursing techniques, provided monitoring and feedback and participated in case study presentations. Simultaneously, they were also required to manage their primary task of caring for patients. Therefore, they require increased coordination with schools.

*‘We need active support from both the teachers and the preceptors’. (Student, R2-S6)*

*‘Because of so many patients and plenty of work to do in the hospital, we must first address our main core tasks (caring for patients). So, our preceptors have a limited time to coach and demonstrate to the students’. (Preceptor, R2-H4)*

### Satisfaction of students with the programme and nursing competency development

The quantitative findings indicated that the satisfaction of the students with the programme was positively correlated with high levels of competency. The qualitative data expanded, providing a broader but overlapping understanding that the students reported two significant inconsistencies between theory and practice.

The first inconsistency was the burden of following double standards in clinical nursing techniques. They learned and observed the latest standard clinical nursing skills in clinical practicum. At the same time, they had to learn different protocols for the same clinical nursing skills at a skills laboratory to prepare for observational structured clinical examinations (OSCE) given as exit examinations in educational institutions.

*‘Sometimes, we had to practice clinical skills which we have not learned at school yet’.* (Student, R2-S6)

*‘Students needed to practice with the mannequins at the school. At the hospital, they had to follow the updated techniques’.* (Preceptor, R3-H2)

Second, the nursing process was partially implemented, and the manner of recording the implementation differed across hospitals. Therefore, in clinical practicum, students could not develop a nursing care plan based on the five steps of the nursing process.

*‘Teachers always asked us to do the nursing process, but at the hospital, we just completed taking vital signs’. (Student, R4-S2)*

*‘In the nursing records at the hospital, ‘planning’ and ‘implementation’ steps were combined. However, students did not recognise that the two steps were already combined’.* (Faculty, R1-S4)

## Discussion

This study integrated quantitative and qualitative findings related to the competency development of the students. The results confirmed each other regarding the effect of CBL on improving nursing competencies. The findings complemented the notion that the teaching by the faculty members and preceptors during clinical practicum improved nursing competencies. Finally, the findings expanded on the inconsistencies between theory and practice in the programme, which may limit the competencies of the students.

### CBL experience and nursing competency development

More than 40% of the students experienced CBL in the classroom and clinical practicum. However, the current curriculum does not mention CBL as a teaching method [32]. CBL experiences facilitated students in gaining an in-depth understanding of professional application of the nursing process in the classroom and the meaning of holistic care through communication and contact with the patients during clinical practicum. Qi et al. [33] performed an experimental study in China and reported that CBL in the classroom promoted the understanding of biomedical subjects (e.g., pathology), professional attitudes, communication skills, and teamwork. Moreover, CBL experiences in the classroom increased the self-efficacy of students in addressing problems in the real-world setting [34]. Meanwhile, Kong et al. [6] conducted a systematic review of CBL in classroom and clinical practicum and suggested that CBL exerted a more positive effect on critical thinking competency than lecture-based teaching. Another CBL experience in clinical practicum helped to develop a professional identity [35]. The results of the current study support the idea that CBL experience in the classroom and clinical practicum is associated with nursing competencies.

Furthermore, small groups that implement CBL in the classroom and clinical settings were culturally appropriate given the seniority culture in Cambodia [36]. Small groups functioned as peer support, which mitigated the negative experiences of students, such as workload, unsupportive work environment and encounter with critically ill patients and their families. In recent years, scholars have re-evaluated peer support in clinical education to reduce challenges, stress and anxiety for students during clinical practicum even in resource-rich settings [37, 38]. Therefore, the results of the current study implied the effectiveness of CBL with peer support in resource-limited settings.

### The role of faculty members and preceptors in nursing competency development

Students who reported high levels of satisfaction with the teaching by faculty members exhibited high levels of nursing competencies. Furthermore, students required additional active support from faculty members and preceptors when faced with various stressors during the clinical practicum. The students emphasised the attributes of interpersonal relationships, such as treating students equally in a culturally appropriate manner and providing more feedback and reflection during clinical practicum [39, 40]. Therefore, the results support the need for the more active involvement of faculty members in clinical practicum.

Preceptors were more involved in the follow-up of students during the clinical practicum, although the students expected more involvement from faculty members. Jayasekara et al. [41] conducted a systematic review and proposed that the clinical educator model is preferable over the preceptor model. In other words, faculty members designated for clinical teaching collaborate with preceptors to coordinate and mentor students during clinical practicum [42]. Students highly appreciated that their clinical instructors visited their clinical sites and bridged the gap between theory and practice [43]. However, these results were obtained in resource-rich settings. Alternatively, the majority of clinical teaching is hospital-oriented education in resource-limited settings [5], and students function as substitutes for human resources. Despite the emergence of innovative methods, such as e-learning [44] and simulation education [45], clinical models that foster the three learning areas, namely, cognitive, psychomotor and affective, remain lacking [46]. Moreover, the application of digital education became increasingly common during the COVID-19 pandemic [47], but scholars have reported constraints on acquiring online clinical skills [48]. This finding suggests that there the need emerges to seek an appropriate clinical teaching model for competency-based education in resource-limited settings.

### Practice and theory gaps in the programme and nursing competency development

This study identified two inconsistencies that may limit the competency development of the students, namely, clinical nursing techniques and limited implementation of the nursing process in clinical settings.

First, students were required to follow double-standard clinical nursing techniques at educational institutions and clinical settings. In Cambodia, students learn and practice updated clinical nursing techniques in clinical practicum. However, they may need to learn outdated standards for the same skills and techniques at educational institutions to prepare for the OSCE as an exit examination, which is nationally standardised by the CMOH. Currently, educational institutions are under pressure to make their students pass licensure examinations [49]. However, scholars reported that an extreme focus on exams may lead students to focus on memorisation instead of the utilisation of critical thinking competencies [50]. Therefore, the results suggest the need for consistency in acquiring nursing techniques in educational institutions and clinical settings.

Second, students learned the five steps of the nursing process according to theory. However, the nursing records in clinical settings endorsed by the CMOH did not cover these five steps. Consequently, students, faculty members and preceptors focus more on the first two steps of the nursing process, namely, assessment and diagnosis. In addition, the implementation of nursing care for non-invasive medical care, the maintenance of personal hygiene and supporting activities of daily living is frequently reliant on family support in Cambodia [51]. Moreover, the limited implementation of the nursing process has also been reported in African and Middle Eastern countries [52]. The factors that aggravate this situation include the lack of understanding by nurses [53], inappropriate ratio of nurses to patients [54] and the limited recognition of the nursing process by other health professionals [55]. Therefore, the limited practice of the nursing process and inappropriate nursing records in clinical settings suggest that the learning of students was not being applied to practice in Cambodia.

Moreover, this study found that both inconsistencies were related to the policy direction established by distinct responsible departments in the CMOH. In resource-limited settings, where the leadership and governance capacity of nurses are limited [56], policy level gaps may be related to the support of development partners [57]. The results suggested the potential need to fill the gap between theory and practice by implementing competency-based education in Cambodia through partnerships between educational institutions and hospitals with strategic policy involvement.

### Limitations

First, the initial research plan included three private educational institutions with more than 20 students located in the capital city, Phnom Penh. However, only two private educational institutions participated in the study. Therefore, this study could only partially assess students and faculty members at private educational institutions, whose teaching and learning environments may have varied.

Second, the study included several clinical practicum sites. The clinical practicum focused only on hospitals, which excluded community nursing practicum sites (e.g., health centres). Moreover, preceptors at health centres were excluded as participants in the FGDs, and the students were not asked questions about their experiences in community nursing practicum. Therefore, the study was unable to assess teaching and learning activities and challenges at the health centre and community levels.

Third, the measurement of competency lacked objectiveness due to self-assessment. To measure competency in the cognitive domain, a knowledge quiz was developed for the nursing process to obtain secondary outcomes. However, it led a methodological error, because the quiz asked for the definitions of each step in the nursing process, instead of examining the utilisation of the nursing process. The OSCE was conducted at each educational institution for the objective evaluation of competency in the psychomotor domain. However, the data of the participants in the current study could not be matched due to the timing and procedure.

Finally, we found that the existing CBL experiences of small groups in the classroom and clinical practicum were associated with the nursing competencies of students, However, this study was unable to compare other qualitative differences in the learning experiences of the students.

## Conclusion

This study was the first to explore the experiences and perceptions of students, faculty members and preceptors regarding the competency development of nursing students in Cambodia. The finding of CBL implementation in small groups in the classroom and clinical settings and the satisfaction of the students with teaching by the faculty members and preceptors improved the competency development of the nursing students. Meanwhile, the satisfaction of the students with the design and delivery of the educational programme provided implications at the policy level to narrow the gaps in theory and practice.

### Electronic supplementary material

Below is the link to the electronic supplementary material.

**Supplementary Material 1: Figure 1**. Recruitment flow for educational institutions

**Supplementary Material 2: Figure 2**. Flow chart of translation and pilot-testing of the questionnaire

**Supplementary Material 3: Table 1**. Complementary quantitative and qualitative data in assessment tools

**Supplementary Material 4: Table 2**. Questionnaire for nursing students

**Supplementary Material 5: Table 3**. FGD topic guide for nursing students, faculty members and preceptors

**Supplementary Material 6: Table 4**. The characteristics of the participants in Focus Group Discussions

## Data Availability

The datasets used and/or analysed during the current study available from the corresponding author on reasonable request. However, due to the sensitive nature of the questions asked in this study, study participants were assured raw data would remain confidential and would not be shared.

## List of abbreviations

ASEAN: Association of Southeast Asian Nations
CBL: Case-Based Learning
CMOH: Cambodian Ministry of Health
FGD: Focus Group Discussion
OSCE: Observational Structured Clinical Examinations
UNSASS: Undergraduate Nursing Student Academic Satisfaction Scale
