# Supplementary figures and images for: Understanding competency of nursing students in the course of case-based learning in Cambodia: a convergent mixed method study
Source: BMC Nurs. 2023 Aug 11;22:265. doi: 10.1186/s12912-023-01420-8 (PMC10416455; doi:10.1186/s12912-023-01420-8)

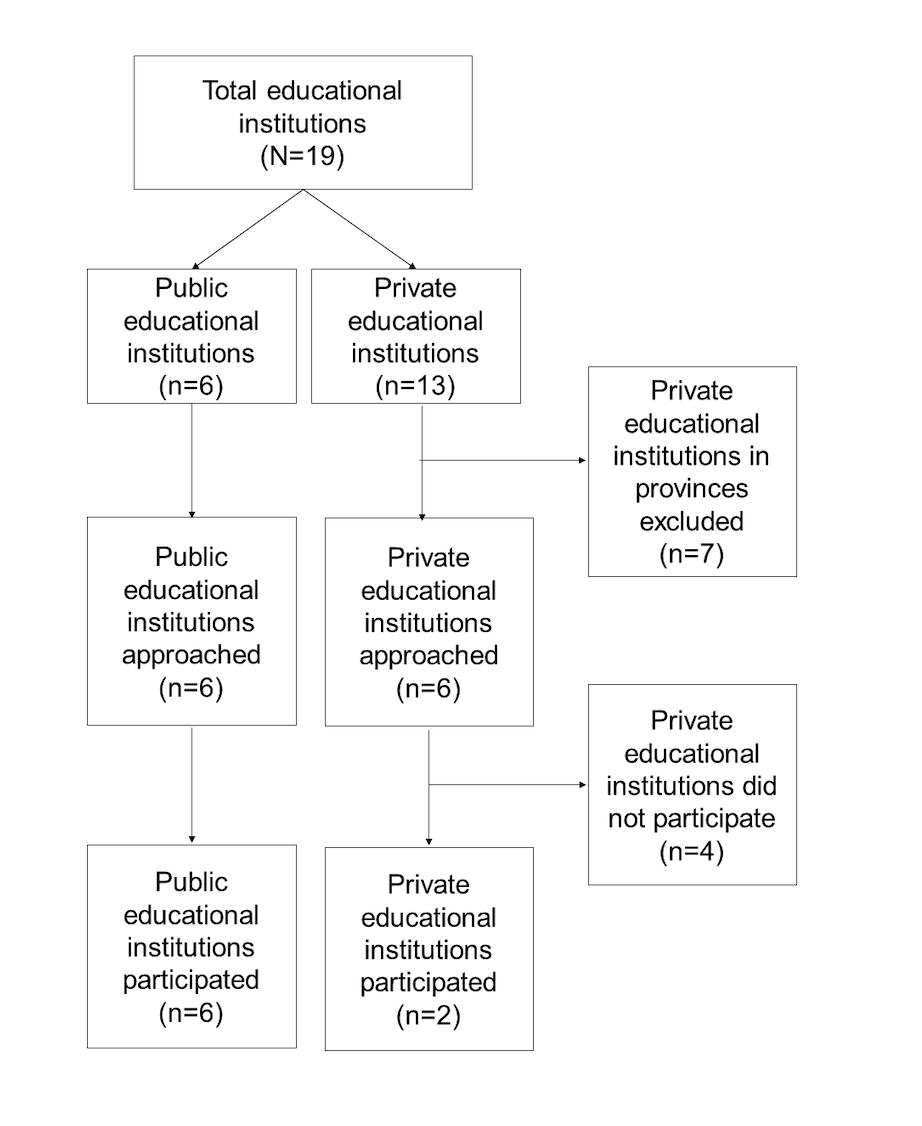

Supplement: Supplementary file 1 — Supplementary Material 1: Figure 1. Recruitment flow for educational institutions [file 12912_2023_1420_MOESM1_ESM.png]

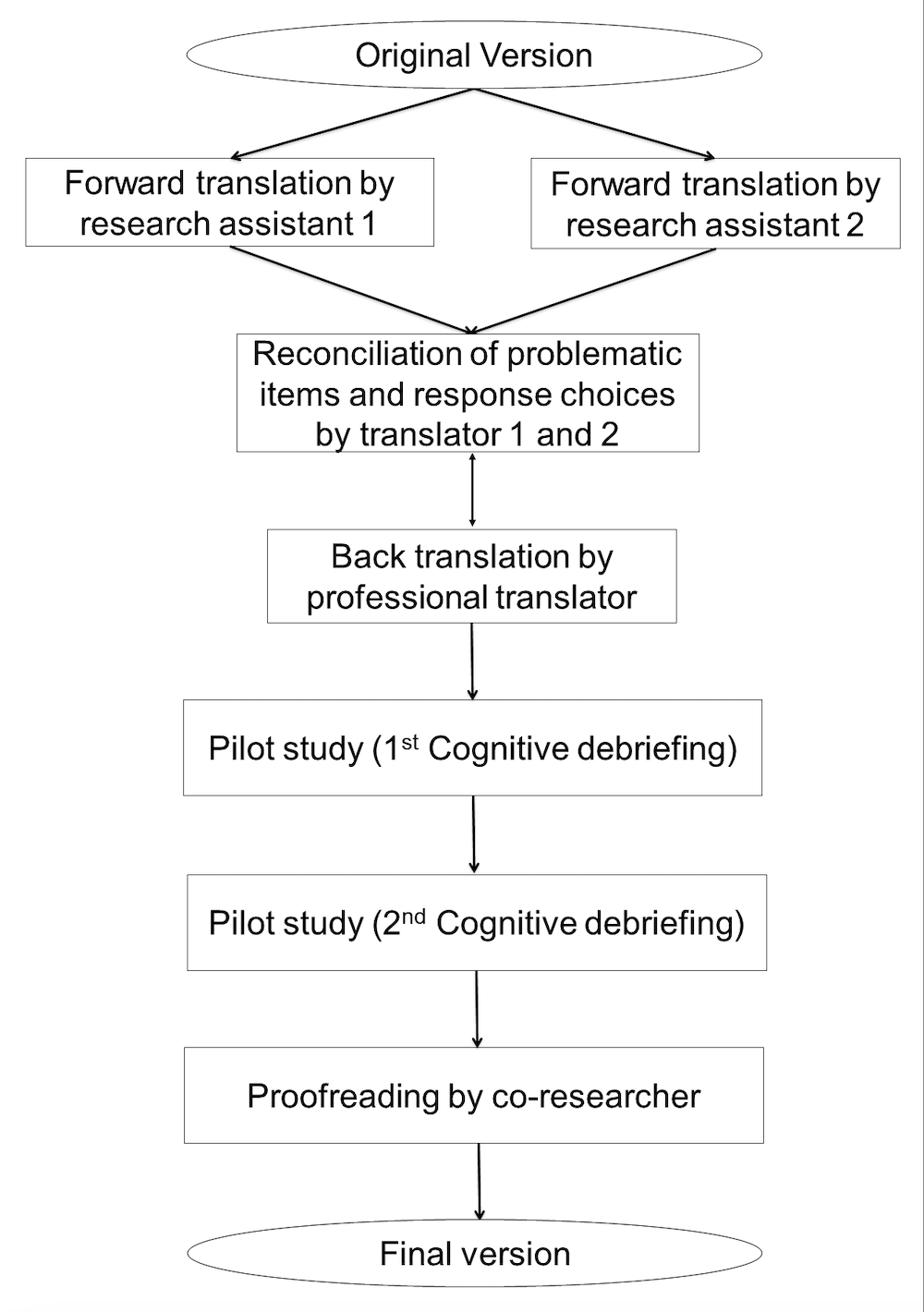

Supplement: Supplementary file 2 — Supplementary Material 2: Figure 2. Flow chart of translation and pilot-testing of the questionnaire [file 12912_2023_1420_MOESM2_ESM.png]
