# Supplementary material for: Understanding competency of nursing students in the course of case-based learning in Cambodia: a convergent mixed method study
Source: BMC Nurs. 2023 Aug 11;22:265. doi: 10.1186/s12912-023-01420-8 (PMC10416455; doi:10.1186/s12912-023-01420-8)
Supplement: Supplementary file 3 — Supplementary Material 3: Table 1. Complementary quantitative and qualitative data in assessment tools [file 12912_2023_1420_MOESM3_ESM.docx]

| **Quantitative data** | | **Qualitative data** | |
| --- | --- | --- | --- |
| **Outcome** | **Exposure** | **Students** | **Faculty members and preceptors** |
| Nursing competency | CBL | Learning nursing process at classroom and clinical practicum  Acquiring nursing competencies | Teaching and learning activities in classroom and clinical practicum |
|  | Support (faculty members and preceptors) | Support by faculty and preceptors |  |
|  | The programme (design, delivery) | Challenges and suggestions for improving teaching and learning | Challenges and suggestions to fulfil gaps between current curriculum and required nursing competencies |
