# Supplementary material for: Understanding competency of nursing students in the course of case-based learning in Cambodia: a convergent mixed method study
Source: BMC Nurs. 2023 Aug 11;22:265. doi: 10.1186/s12912-023-01420-8 (PMC10416455; doi:10.1186/s12912-023-01420-8)
Supplement: Supplementary file 4 — Supplementary Material 4: Table 2. Questionnaire for nursing students [file 12912_2023_1420_MOESM4_ESM.docx]

Serial #:

Assessment of nursing competency among nursing students in Cambodia

This questionnaire will assess your own daily learning and use it to improve the nursing curriculum and improve the learning environment for nursing student. It will never lead to the evaluation of your own grades. If there is anything you do not understand in the question item, please contact the researcher in the place.

Please circle on one for options in number and more than one for options in alphabet.

**Socio-demographic characteristics**

1. Age: years old
2. Sex:
   - 1. Male
     2. Female
3. Type of school and course:
   - 1. Bachelor of Science in Nursing (BSN) @Technical School of Medical Care
     2. Associate Degree of Nursing (ADN) @ Technical School of Medical Care
     3. RTC (Name of School: )
     4. Bachelor of Science in Nursing (BSN) @ Private university (Name of School: )
     5. Associate Degree of Nursing (ADN) @ Private university (Name of School: )
4. Please specify your high school grade from A to E: ___________
5. English proficiency:
6. Very high
7. High
8. Moderate
9. Low
10. Very low
11. Previous working experience before studying in nursing programe: month(s) in total (Please specific type of work: )

If you have no experience of working, please write 0 in column above.

1. What would you like to do after completing the current study? (You can select more than one)
2. Work as nurse at public hospital or health center
3. Work as nurse at public health administration (e.g. Ministry, Provincial and or operational health district)
4. Work as nurse at private hospital or clinic
5. Work as nurse at school as teacher (Public school or Private school)
6. Work in health sector, but not as nurse
7. Work, but not in health sector
8. Continue study nursing (Bachelor degree or Master degree)
9. Continue study other than nursing in health (Medicine, Dentist, Pharmacist, Public health)
10. Continue study other than health (e.g. Business)
11. Others:
12. Which step of nursing process is most important for you ?
13. Assessment
14. Diagnosis
15. Planning
16. Implementation
17. Evaluation
18. Which step of nursing process is most difficult for you to understand?
19. Assessment
20. Diagnosis
21. Planning
22. Implementation
23. Evaluation
24. Have you learnt nursing process by case scenario at school?
25. Yes

In which subjects? e.g. Adult Nursing

1. No
2. Don’t know
3. Please write down your information about experience on clinical practice from year 1 to year 3.

| Hospital | Department | Length of practice in weeks or months | Did you apply nursing process through case study/assignment? |
| --- | --- | --- | --- |
| E.g. Khmer Soviet Friendship Hospital | General medicine | 3 weeks | 1. Yes 2. No 3. Don’t know |
|  |  |  | 1. Yes 2. No 3. Don’t know |
|  |  |  | 1. Yes 2. No 3. Don’t know |
|  |  |  | 1. Yes 2. No 3. Don’t know |
|  |  |  | 1. Yes 2. No 3. Don’t know |
|  |  |  | 1. Yes 2. No 3. Don’t know |
|  |  |  | 1. Yes 2. No 3. Don’t know |

**General Self-Efficacy Scale**

Each statement below describes your characteristic.

For each item, please indicate the extent to which you agree that the item is YOUR CHARACTERISTIC from 1 (Not at all true) to 4(Exactly true).

|  | **Not at all true**  **1** | **Hardly true**  **2** | **Moderately true**  **3** | **Exactly true**  **4** |
| --- | --- | --- | --- | --- |
| 1. I can always manage to solve difficult problems if I try hard enough | **1** | **2** | **3** | **4** |
| 2. If someone opposes me, I can find the means and ways to get what I want. | **1** | **2** | **3** | **4** |
| 3. It is easy for me to stick to my aims and accomplish my goals. | **1** | **2** | **3** | **4** |
| 4. I am confident that I could deal efficiently with unexpected events. | **1** | **2** | **3** | **4** |
| 5. Thanks to my problem solving abilitiy, I know how to handle unforeseen situations. | **1** | **2** | **3** | **4** |
| 6. I can solve most problems if I invest the necessary effort. | **1** | **2** | **3** | **4** |
| 7. I can remain calm when facing difficulties because I can rely on my coping abilities. | **1** | **2** | **3** | **4** |
| 8. When I am confronted with a problem, I can usually find several solutions. | **1** | **2** | **3** | **4** |
| 9. If I am in trouble, I can usually think of a solution. | **1** | **2** | **3** | **4** |
| 10. I can usually handle whatever comes my way. | **1** | **2** | **3** | **4** |

**Competency Inventory for Nursing Student**

Each statement below describes your level of frequency in nursing practice.

For each item, please indicate how often you perform, from 1 (Not at all) to 7 (always) in your current nursing study.

|  |  | **1**  **Not at all** | **2**  **Seldom** | **3**  **Occasionally** | **4**  **Sometimes** | **5**  **Frequently** | **6**  **Nearly always** | **7**  **Always** |
| --- | --- | --- | --- | --- | --- | --- | --- | --- |
| 1 | I make use of knowledge of anatomy and human pathology to explain to patients their conditions. | 1 | 2 | 3 | 4 | 5 | 6 | 7 |
| 2 | I explain to patients the procedure of physical examinations. | 1 | 2 | 3 | 4 | 5 | 6 | 7 |
| 3 | I understand the indication and side effects of the medication that patients are prescribed. | 1 | 2 | 3 | 4 | 5 | 6 | 7 |
| 4 | I understand the purposes and normal values of clinical examinations. | 1 | 2 | 3 | 4 | 5 | 6 | 7 |
| 5 | I understand the rationale for the choice of medical treatment. | 1 | 2 | 3 | 4 | 5 | 6 | 7 |
| 6 | I carry out doctor's instructions and record accurately. | 1 | 2 | 3 | 4 | 5 | 6 | 7 |
| 7 | I collect as much information about the patient as possible to formulate the best nursing care plan. | 1 | 2 | 3 | 4 | 5 | 6 | 7 |
| 8 | I assess a patient's needs for nursing implementation based on data available to me. | 1 | 2 | 3 | 4 | 5 | 6 | 7 |
| 9 | I provide patients with nursing treatments that suit their needs. | 1 | 2 | 3 | 4 | 5 | 6 | 7 |
| 10 | I evaluate the outcome of my nursing implementation. | 1 | 2 | 3 | 4 | 5 | 6 | 7 |
| 11 | I set priorities of nursing diagnosis based on the needs of patients. | 1 | 2 | 3 | 4 | 5 | 6 | 7 |
| 12 | I try to provide comprehensive follow-up of nursing care that suits the needs of patients. | 1 | 2 | 3 | 4 | 5 | 6 | 7 |
| 13 | I record nursing care plan accurately. | 1 | 2 | 3 | 4 | 5 | 6 | 7 |
| 14 | I carefully consider the condition of patients and make reasonable judgments. | 1 | 2 | 3 | 4 | 5 | 6 | 7 |
| 15 | I try to assess the reason of any changes in patients' conditions. | 1 | 2 | 3 | 4 | 5 | 6 | 7 |
| 16 | I always think about whether there is reference to support nursing actions. | 1 | 2 | 3 | 4 | 5 | 6 | 7 |
| 17 | I try to compassionate with patients in order to identify their needs for nursing care. | 1 | 2 | 3 | 4 | 5 | 6 | 7 |
| 18 | I encourage patients to express their positive and negative emotions about how they feels of their illness and treatment. | 1 | 2 | 3 | 4 | 5 | 6 | 7 |
| 19 | I provide timely emotional support for patients when necessary. | 1 | 2 | 3 | 4 | 5 | 6 | 7 |
| 20 | I pay attention to the psychological and social wellbeing of patients. | 1 | 2 | 3 | 4 | 5 | 6 | 7 |
| 21 | I try my best to provide patients with a comfortable and peaceful environment. | 1 | 2 | 3 | 4 | 5 | 6 | 7 |
| 22 | I believe that each patient's life has it own values. | 1 | 2 | 3 | 4 | 5 | 6 | 7 |
| 23 | I abide by the codes of nursing ethics and other related rules and regulations. | 1 | 2 | 3 | 4 | 5 | 6 | 7 |
| 24 | I try my best to keep patients from harm in providing nursing care. | 1 | 2 | 3 | 4 | 5 | 6 | 7 |
| 25 | I am always careful not to infringe upon patients' rights when making decisions concerning patients. | 1 | 2 | 3 | 4 | 5 | 6 | 7 |
| 26 | I have respect for the decisions and choices of patients. | 1 | 2 | 3 | 4 | 5 | 6 | 7 |
| 27 | I respect patients' confidentiality nursing | 1 | 2 | 3 | 4 | 5 | 6 | 7 |
| 28 | I provide patients with safe nursing care. | 1 | 2 | 3 | 4 | 5 | 6 | 7 |
| 29 | I have full respect for the different beliefs and values about treatment held by patients (e.g. Using of traditional healer and medicine) . | 1 | 2 | 3 | 4 | 5 | 6 | 7 |
| 30 | I actively fulfill my duties. | 1 | 2 | 3 | 4 | 5 | 6 | 7 |
| 31 | I am accountable for my professional judgments and actions. | 1 | 2 | 3 | 4 | 5 | 6 | 7 |
| 32 | I am aware of the limitations of my professional role and responsibilities. | 1 | 2 | 3 | 4 | 5 | 6 | 7 |
| 33 | I consult to senior nurses when I come across a situation that is beyond my ability to handle. | 1 | 2 | 3 | 4 | 5 | 6 | 7 |
| 34 | I take my nursing work seriously and conduct my nursing work carefully. | 1 | 2 | 3 | 4 | 5 | 6 | 7 |
| 35 | I make effective use of my time at work. | 1 | 2 | 3 | 4 | 5 | 6 | 7 |
| 36 | I am aware of what I need to learn. | 1 | 2 | 3 | 4 | 5 | 6 | 7 |
| 37 | I enjoy seeking answers to questions. | 1 | 2 | 3 | 4 | 5 | 6 | 7 |
| 38 | I set goals for my learning. | 1 | 2 | 3 | 4 | 5 | 6 | 7 |
| 39 | I know where and how to look for learning documents for learning. | 1 | 2 | 3 | 4 | 5 | 6 | 7 |
| 40 | I make use of internet or e-learning for my study. | 1 | 2 | 3 | 4 | 5 | 6 | 7 |

**Undergraduate Nursing Student Academic Satisfaction Scale**

Each statement below describes your experience at your school. Please read each statement carefully; then rate your agreement level in terms of 1=*Strongly disagree* to 5=*Strongly agree* for each statement.

|  |  | **1**  **Strongly disagree** | **2**  **Disagree** | **3**  **Somewhat agree** | **4**  **Agree** | **5**  **Strongly agree** |
| --- | --- | --- | --- | --- | --- | --- |
| 1 | I can freely express my academic and other concerns to lecturers. | 1 | 2 | 3 | 4 | 5 |
| 2 | Lecturers are easily approachable. | 1 | 2 | 3 | 4 | 5 |
| 3 | Lecturers make every effort tot help students when asked. | 1 | 2 | 3 | 4 | 5 |
| 4 | Lecturers make an effort to understand student’s difficulties. | 1 | 2 | 3 | 4 | 5 |
| 5 | Lecturers are available after class for questions | 1 | 2 | 3 | 4 | 5 |
| 6 | I can freely express my concerns to training unit for nurses. | 1 | 2 | 3 | 4 | 5 |
| 7 | Lecturers are fair and unbiased with students. | 1 | 2 | 3 | 4 | 5 |
| 8 | Lecturers give students adequate feedback on their progress. | 1 | 2 | 3 | 4 | 5 |
| 9 | Lecturers effectively explained essential concepts. | 1 | 2 | 3 | 4 | 5 |
| 10 | Students know who to go if they have complaints. | 1 | 2 | 3 | 4 | 5 |
| 11 | Lecturers are good role models and motivate me to do my best. | 1 | 2 | 3 | 4 | 5 |
| 12 | Training unit for nurses shows concern for students. | 1 | 2 | 3 | 4 | 5 |
| 13 | Lecturers demonstrate a high level of knowledge in their subject. | 1 | 2 | 3 | 4 | 5 |
| 14 | Lecturers take the time to listen students about academic issues. | 1 | 2 | 3 | 4 | 5 |
| 15 | Lecturers create a good overall impression. | 1 | 2 | 3 | 4 | 5 |
| 16 | I am generally given enough time to understand the things I have to learn | 1 | 2 | 3 | 4 | 5 |
| 17 | Preceptors are approachable and students can ask questions. | 1 | 2 | 3 | 4 | 5 |
| 18 | Preceptors provide feedback at appropriate times. | 1 | 2 | 3 | 4 | 5 |
| 19 | Preceptors are open to discussion of procedures. | 1 | 2 | 3 | 4 | 5 |
| 20 | Preceptors give me sufficient guidance before I perform a procedure. | 1 | 2 | 3 | 4 | 5 |
| 21 | Preceptors help me learn from my mistake. | 1 | 2 | 3 | 4 | 5 |
| 22 | Preceptors give me clear information of what is expected when going to practicum. | 1 | 2 | 3 | 4 | 5 |
| 23 | Preceptors help me develop critical thinking skills in my nursing practice. | 1 | 2 | 3 | 4 | 5 |
| 24 | Preceptors teach me about patient care and nursing process when I am in practicum. | 1 | 2 | 3 | 4 | 5 |
| 25 | I get adequate feedback to improve my skills when I am in practicum. | 1 | 2 | 3 | 4 | 5 |
| 26 | Preceptors demonstrate a high level of knowledge. | 1 | 2 | 3 | 4 | 5 |
| 27 | Preceptors are usually available for students in practicum. | 1 | 2 | 3 | 4 | 5 |
| 28 | Preceptors provide enough opportunities for self practice in the hospital. | 1 | 2 | 3 | 4 | 5 |
| 29 | Preceptors encourage me to link theory to practice. | 1 | 2 | 3 | 4 | 5 |
| 30 | Preceptors teach procedures the same as practiced in skill lab. | 1 | 2 | 3 | 4 | 5 |
| 31 | The nursing teachers and preceptors collaboratively worked with each other in their teaching. | 1 | 2 | 3 | 4 | 5 |
| 32 | The nursing program provides a variety of good and relevant courses. | 1 | 2 | 3 | 4 | 5 |
| 33 | The nursing program enhances my skill to utilize the nursing process in my clinical practice. | 1 | 2 | 3 | 4 | 5 |
| 34 | Most courses in the nursing program are beneficial for my overall capacity development in nursing. | 1 | 2 | 3 | 4 | 5 |
| 35 | The course outline/lesson plan clearly described what was expected of me in each nursing subject. | 1 | 2 | 3 | 4 | 5 |
| 36 | I usually have a clear idea of what is expected of me in the nursing program. | 1 | 2 | 3 | 4 | 5 |
| 37 | The nursing program helps students learn teamwork. | 1 | 2 | 3 | 4 | 5 |
| 38 | The nursing program helps my problem solving and decision making skills | 1 | 2 | 3 | 4 | 5 |
| 39 | There is a commitment to academic quality in the nursing school. | 1 | 2 | 3 | 4 | 5 |
| 40 | As a results of my learning at classroom and clinical, I feel confident in dealing with clinical nursing problems. | 1 | 2 | 3 | 4 | 5 |
| 41 | Going to class helps me understand the lesson. | 1 | 2 | 3 | 4 | 5 |
| 42 | Overall, the nursing program requirements are reasonable and achievable | 1 | 2 | 3 | 4 | 5 |
| 43 | There was sufficient equipment in the nursing lab for my learning. | 1 | 2 | 3 | 4 | 5 |
| 44 | The library resources were adequate for my learning. | 1 | 2 | 3 | 4 | 5 |

*Thank you very much for your cooperation.*
