# Supplementary material for: Understanding competency of nursing students in the course of case-based learning in Cambodia: a convergent mixed method study
Source: BMC Nurs. 2023 Aug 11;22:265. doi: 10.1186/s12912-023-01420-8 (PMC10416455; doi:10.1186/s12912-023-01420-8)
Supplement: Supplementary file 5 — Supplementary Material 5: Table 3. FGD topic guide for nursing students, faculty members and preceptors [file 12912_2023_1420_MOESM5_ESM.docx]

| **Nursing student** | **Faculty member and preceptor** |
| --- | --- |
| **Experience of learning on nursing process:** | **Current teaching-learning activities for establishing critical thinking requirements:** |
| How do you evaluate yourself in terms of understanding nursing process in lecture and clinical setting? | How did you learn/experience on utilization of nursing process? |
| How did you learn the nursing process at classroom setting? Please explain about one case. Which form/document did you utilize? *(by showing MoH form and logbook)* | In which subjects do you teach nursing process? |
| And how did you apply the nursing process at clinical setting? Please explain about one case. Which form/document did you utilize? *(by showing MoH form and logbook)* | How do you teach nursing process at a classroom setting (e.g. case scenario)? Have you utilized the manual of nursing process by MoH? |
| Many students answered that the most important step in the nursing process is assessment. But, many students answered that most difficult steps in the nursing process to apply are diagnosis, planning and evaluation? Why’s that so? | Which nursing theory do you utilize in teaching nursing process? |
| What kind of challenges did you face in learning on the nursing process at classroom setting and at clinical setting? How do you apply biomedical knowledge (e.g. anatomy and physiology) into nursing practice? | How do you assess students’ learning/understanding on the nursing process (e.g. assignment, group presentation, mid-term, final exam)? |
| How did you overcome these challenges? When you have difficulty in learning, how do you solve the problem? | What do you think are the most important steps of the nursing process a student need to acquire? Which steps of the nursing process are difficult for nursing students to achieve? |
| When you face the patients in need of emotional /psychological support, what did you do? | According to students, the most important step is “assessment”. And they feel difficulty in “diagnosis”, “planning” and “evaluation”. What do you think of the gap? |
| What kind of support/guidance did the preceptors provide? How did you feel about it? What kind of support/guidance did you expect preceptors to provide? Which hospital/ward was helpful for learning (conduct case study)? | What kind of challenges/difficulties do you face in current teaching-learning activities on nursing process? How do you overcome these challenges? How students approach to teachers when they have difficulties in learning? |
| What kind of support/guidance did instructors provide? How did you feel about it? What kind of support/guidance did you expect instructors to provide? | How do you perceive the role of the nursing teachers and clinical preceptors in clinical practice? |
| When you have problem in learning at school, to whom you approach? | Which part of the curriculum needs to be improved to meet the critical thinking requirements? How teachers and clinical preceptors collaboration can be improved? |
| How do you think/What is you opinion about clinical practices can be improved? |  |
| **Motive as nursing student** | **Current teaching-learning environment** |
| How do you feel about being a student in nursing education? Who is your role model as nurse? | Number of nursing students has been decreasing since 2013. What are the factors? How do you think about this? How does it affect to teaching-learning at school and clinical setting? |
| After the graduation, where would you like to work or study? Why? | What recommendation or suggestions to Ministry to tackle this issue? |

*Thank you very much.*
