# Supplementary material for: Understanding competency of nursing students in the course of case-based learning in Cambodia: a convergent mixed method study
Source: BMC Nurs. 2023 Aug 11;22:265. doi: 10.1186/s12912-023-01420-8 (PMC10416455; doi:10.1186/s12912-023-01420-8)
Supplement: Supplementary file 6 — Supplementary Material 6: Table 4. The characteristics of the participants in Focus Group Discussions [file 12912_2023_1420_MOESM6_ESM.docx]

| **Type of participants** | **Average age (range)** | **Sex** | **Years of experience as nurse** | **Years of experience as faculty/preceptor** |
| --- | --- | --- | --- | --- |
| Faculty members (n=38) | 38.1 years  (24–63) | 42% women | 13.1 years^[[1]](#footnote-1)^∗  (4–25) | 11.6 years  (3–30) |
| Preceptors  (n=37) | 40.2 years  (26–60) | 38% women | 20.9 years  (4–34) | 7.9 years  (1–15) |
| Students  (n=55) | 22.4 years  (19–29) | 50% women | - | - |

1. ^∗^ Excluded five faculties who are medical doctors. [↑](#footnote-ref-1)
